# The Prevalence of Menstrual Cycle Disorders in Female Athletes from Different Sports Disciplines: A Rapid Review

**DOI:** 10.3390/ijerph192114243

**Published:** 2022-10-31

**Authors:** Marta Gimunová, Alexandra Paulínyová, Martina Bernaciková, Ana Carolina Paludo

**Affiliations:** 1 Department of Kinesiology, Faculty of Sport Studies, Masaryk University, 62500 Brno, Czech Republic; 2 Incubator of Kinanthropological Research, Faculty of Sport Studies, Masaryk University, 62500 Brno, Czech Republic

**Keywords:** menstrual disorders, oligomenorrhea, amenorrhea, sports training, female athlete, Olympic sports

## Abstract

The aim of this study was to rapidly review the literature on the prevalence of menstrual disorders in female athletes from different sports modalities. Articles were searched in the Web of Science and PubMed database in May 2022. A total of 1309 records were identified, and 48 studies were included in the final stage. The menstrual disorders described in the included studies were primary (in 33% of included studies) and secondary amenorrhea (in 73% of included studies) and oligomenorrhea (in 69% of included studies). The prevalence of menstrual disorders among the studies ranged from 0 to 61%. When data were pooled according to discipline (mean calculation), the highest prevalence of primary amenorrhea was found in rhythmic gymnastics (25%), soccer (20%) and swimming (19%); for secondary amenorrhea in cycling (56%), triathlon (40%) and rhythmic gymnastics (31%); and oligomenorrhea in boxing (55%), rhythmic gymnastics (44%) and artistic gymnastics (32%). Based on the results of this review, the study supports the literature of the higher prevalence of menstrual disorders in gymnastics and endurance disciplines. However, team sports modalities such as volleyball and soccer also presented a considerable percentage of menstrual disorders compared to the general population. It reinforces the importance of coaches and physicians paying attention to athletes’ menstrual cycle as the occurrence of menstrual disorders can be associated with impairment on some health components.

## 1. Introduction

For women involved in sports, the incidence of menstrual irregularities has been reported to be higher compared to the general population, especially at the professional level [1]. High physical demands and insufficient recovery, together with long-term inadequate nutritional intake and psychological stress are potential factors that cause an imbalance in the neuroendocrine process related to the hypothalamic-pituitary-ovarian (HPO) axis, the system controlling female reproduction [2]. The malfunction of the HPO axis can trigger changes in luteinizing hormone pulsatility [3] and estrogen deficiency, leading to a variety of menstrual disturbances such as amenorrhea and oligomenorrhea [4,5].

The definitions of primary and secondary amenorrhea and oligomenorrhea can vary in the scientific literature. Primary amenorrhea is usually defined as a failure to reach the first menstrual period—menarche [6], and secondary amenorrhea is defined as the absence of menstruation for 3 or more months in women with previously regular menses or for 6 months in women with previously irregular menses [7,8]. Functional hypothalamic amenorrhea (FHA) is one of the main causes of secondary amenorrhea and it is characterized by the suppression of the HPO axis [9]. Three types of FHA have been recognized: weight loss-related, stress-related, and exercise-related amenorrhea [9,10], and their long-term consequences include infertility, delayed puberty, deficiency in bone mineral density, or cardiovascular consequences [11,12,13]. Oligomenorrhea is defined as irregular and prolonged menstrual blood flow, greater than 35 days apart or a total of 5 to 7 cycles a year [14,15]. The main causes of oligomenorrhea are dysfunctions of the HPO axis that in long-term can lead to several complications, such as infertility, hirsutism, low bone density, and endometrial or breast cancer [15,16].

These menstrual disorders aforementioned have been considered one of the negative components of the Relative Energy Deficiency in Sport (RED-s), introduced recently by the International Olympic Committee, beyond what is known as the female athlete triad [17]. The occurrence of RED-s and menstrual disorders are more likely to emerge in sports that require low weight such as ballet dancing, gymnastics, figure skating and runners [18,19]. Indeed, higher incidences of menstrual disorders in leanness or in aesthetic sports are widely reported; however, the prevalence in other sports disciplines is still not well documented [2]. The main aim of the study was to rapidly review the literature about the prevalence of menstrual disorders in athletes from different sports disciplines. Second, we aimed to describe the most common disorders according to the sports disciplines. Increasing awareness about the percentage of menstrual cycle disorders among the disciplines may facilitate decision-making based on evidence from those who are involved in female athletes’ training (e.g., coaches, physicians).

## 2. Materials and Methods

The rapid review was proposed as a methodological approach to accelerate the process of the traditional systematic review guidelines. This method helps to produce evidence for stakeholders in a pragmatic shortcut, facilitating the knowledge translation between scientific evidence and practical context, helping the decision-making in health practice [20,21]. In the case of this review, the rapid review approach can help to hasten the knowledge-translation process in order to identify which sports disciplines are more likely to be at risk of menstrual irregularities, providing early diagnosis and intervention to avoid impairment of female reproductive health. The review was performed under the guidelines of the Cochrane Rapid Reviews Guidance [20] together with the Preferred Reporting Items for Systematic Reviews and Meta-Analyses—PRISMA 2020 [22].

### 2.1. Search and Eligibility Criteria

A search was performed using two databases: PubMed and Web of Science in March 2022 by one researcher (AP). The eligibility criteria included: (1) professional female athletes across different sports disciplines included into the Olympic Games [23] and age categories; (2) retrospective and cross-sectional studies; and (3) a description of the prevalence of menstrual irregularities in athletes. The following search terms with Boolean operators were used: (“menstrual cycle disorders” OR “menstrual cycle abnormalities” OR “menstrual cycle disturbances” OR “menstrual cycle disruption” OR “menstrual cycle irregularity” OR “menstrual cycle absence” OR “amenorrhea” OR “oligomenorrhea” OR “abnormal menstrual cycle bleeding” OR “abnormal uterine bleeding” OR “anovulation” OR “dysmenorrhea” OR “premenstrual syndrome” OR “menstrual delay” OR “delayed menstruation” OR “heavy menstrual bleeding” OR “female athlete triad” OR “menorrhagia” OR “excessive uterine bleeding”) AND (“alpine skiing” OR “aquatics” OR “archery” OR “artistic gymnastics” OR “artistic swimming” OR “athletics” OR “badminton” OR “baseball” OR “basketball” OR “beach volleyball” OR “boxing” OR “biathlon” OR “bobsleighing” OR “canoe” OR “cross-country skiing” OR “curling” OR “cycling” OR “diving” OR “equestrian” OR “fencing” OR “figure skating” OR “football” OR “freestyle skiing” OR “golf” OR “gymnastics” OR “handball” OR “hockey” OR “horse riding” OR “ice hockey” OR “judo” OR “karate” OR “kayak” OR “luge” OR “Nordic combined” OR “marathon swimming” OR “mountain bike” OR “pentathlon” OR “rhythmic gymnastics” OR “rugby” OR “running” OR “rowing” OR “sailing” OR “shooting” OR “short track” OR “skateboarding” OR “skeleton” OR “ski jumping” OR “snowboarding” OR “sport climbing” OR “soccer” OR “speed skating” OR “surfing” OR “swimming” OR “table tennis” OR “taekwondo” OR “tennis” OR “track and field” OR “trampoline” OR “triathlon” OR “volleyball” OR “water polo” OR “wrestling” OR “weightlifting” OR “3 × 3 basketball” OR “softball” OR “BMX racing” OR “BMX freestyle” OR “road cycling” OR “track cycling”). It was established that articles published from the year of 2000 until May of 2022 were included in this study. Exclusion criteria included articles with recreational athletes, non-English language, systematic reviews, magazine articles and articles with no full-text available.

### 2.2. Study Selection and Data Extraction

**Figure 1:**
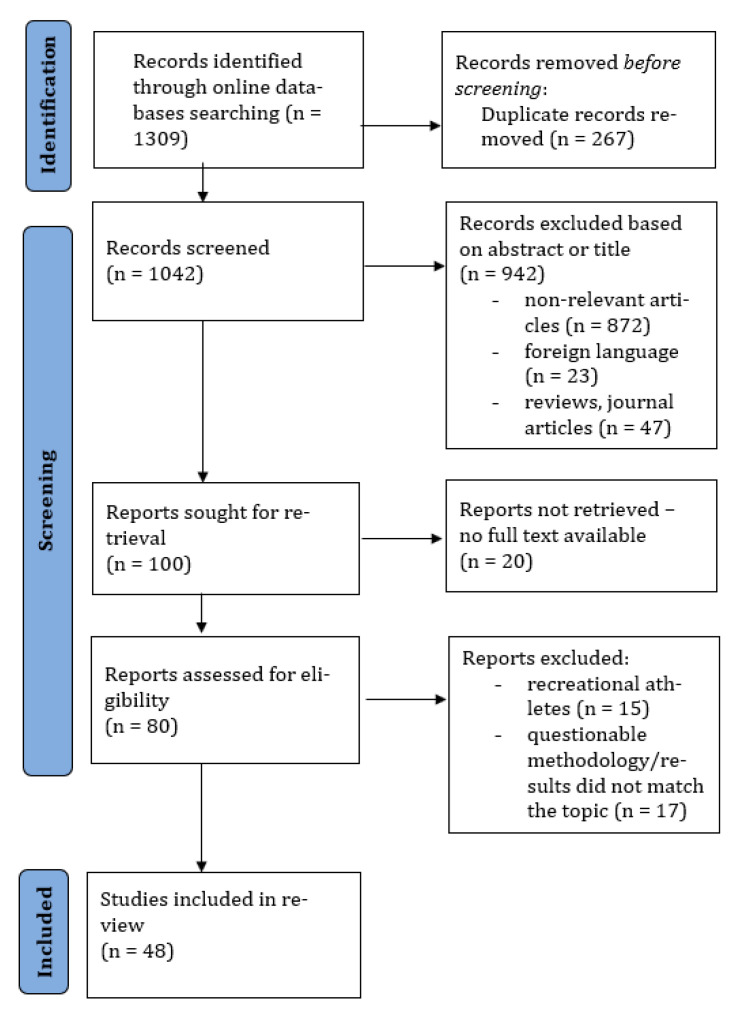
Flow chart diagram of the study process.

After the search, articles were imported into the Rayyan systematic review software [24] by one researcher (AP), and duplicate, reviews and non-English language articles were excluded. Title and abstract were scanned by one researcher (AP) and the excluded articles were verified by a second researcher (ACP). Any disagreement between reviewers was consulted by a third reviewer (MG). Full text and data extraction were performed by two independent reviewers (AP, MG). To extract the data, a form was developed by the researchers with information about the sample characteristics, sports discipline, age of menarche, methods, monitored period, menstrual disorders’ prevalence (primary amenorrhea, secondary amenorrhea, oligomenorrhea, other) and the definition of the menstrual disorder. The process is summarized in the study selection flow chart (Figure 1).

### 2.3. Studies Methodological Quality

The Adjusted Downs and Black Quality Assessment Checklist [25] was used to assess the quality of the studies. The original protocol consists of 27 “yes” or “no” questions divided into five sections: quality of reporting, external validity, internal validity bias, confounding and selection bias, and power of the study [25], with a binary score for each question: 0 = no/unable to determine and 1 = yes. For the present review, it was considered 13 items relevant (Supplementary Materials Table S1). The rating system was adjusted, and the final score was converted to percentages and classified as follows: <45.4% “poor” methodological quality; 45.5–61.0% “fair” methodological quality”; and >61.0% “good” methodological quality [26]. The quality assessment was not used to exclude any study. The same approach was applied in previous review studies, e.g., [26,27,28].

### 2.4. Mean Prevalence Separated by Sports Discipline

To describe the prevalence of menstrual irregularities (primary amenorrhea, secondary amenorrhea and oligomenorrhea) considering each sport discipline, an arithmetic mean, minimum and maximum prevalence (if more than one study was available) were calculated, pooling together the values of all studies that evaluated the same discipline.

## 3. Results

### 3.1. Study Selection

After entering the keywords into the search databases PubMed and Web of Science, 1309 studies were found. Duplicate articles were removed (*n* = 267), and 70 were excluded for presenting a review method or were in a foreign language. The title and abstract were screened in 972 articles, and 872 were excluded. In the last stage, 20 articles were excluded due to no full text being available, 15 articles were excluded to assess recreational athletes and 17 were excluded because the outcomes did not match with the topic. A total of 48 articles were included in the last stage of the review (Figure 1).

### 3.2. Description of Included Studies

The methodological quality of the studies included is summarized in Supplementary Materials (Tables S1 and S2). Most of the studies (*n* = 42) presented a score higher than 61.0%, which classifies them as good quality studies (ranging from 69 to 92%). Six studies were classified as fair quality. The most common methodological deficits in the articles were the lack of representativeness of the source population, reliability of outcome measures, and recruitment of participants over the same period. The disciplines were grouped by categories according to their characteristics [29]: team sports (basketball, field hockey, ice hockey, soccer, softball, synchronized swimming, volleyball, water polo), cyclic sports (running, rowing, swimming, cycling, triathlon, track-and-field), individual sports (boxing, fencing, figure skating, gymnastics, tennis) and winter sports (skiing, biathlon, bobsleigh, luge, skeleton, snowboarding, speed skating). In the case of a study that assessed more than one discipline (e.g., runners and soccer players), each discipline was counted individually.

The majority of the studies focused on runners (*n* = 21), gymnasts (*n* = 14) and track-and-field (*n* = 6) athletes. Included participants varied from elite athletes (e.g., [30,31,32]) to collegiate athletes (e.g., [33,34]). Menstrual disorders investigated in the studies were primary (in 33% of included studies) and secondary amenorrhea (in 73% of included studies), oligomenorrhea (in 69% of included studies) and other menstrual irregularities (in 23% of included studies). The prevalence of menstrual disorders ranges from non-disorder (0%) as observed in water polo players [33] to a maximum percentage of 53.8% of primary, 30.8% of secondary amenorrhea and 61% of oligomenorrhea observed in rhythmic gymnastics athletes [31,35,36]. The menstrual disorder reported in the studies examined a retrospective period varying from the last 3, 6 or 12 months, 5 years or one year after menarche (Table 1).

#### 3.2.1. Team Sports

Fifteen studies reported the prevalence of menstrual disorders in team sports disciplines (Table 2). Menstrual disorders were reported based on the last 12 months [33,34,37], 6 months [38], or in the post-menarche period [39,40]. The highest prevalence of primary amenorrhea (20%) was observed in soccer players [33], secondary amenorrhea (30%) in volleyball players [39], and oligomenorrhea (20%) in soccer players [33]. Up to 71.5% of ice hockey players experienced minor menstrual dysfunction [37].

#### 3.2.2. Cyclic Sports

Third-sixty investigations described the prevalence of menstrual disorders in cyclic sports (Table 3). Menstrual disorders were reported based on the last 5 years [41], 12 months (e.g., [33,34,42]), 6 months [43] or in post-menarche [39,44,45]. In cyclic sports, the highest prevalence of primary amenorrhea (20%) was observed in middle/long distance runners [39], secondary amenorrhea (55% and 55.6%) in middle/long distance runners and cyclists, respectively [39,46]. The highest prevalence of oligomenorrhea (47.5%) was observed in endurance athletes [47]. Menstrual cycle (MC) irregularity was observed in 83.3% of lightweight rowers [45].

#### 3.2.3. Other Individual Sports

Nineteen investigations described menstrual disorders in individual sports (Table 4). Most of the studies focused on rhythmic gymnastics [30,31,35,36,67,68,69,70,71,72], artistic gymnastics [72,73] and gymnastics [33,34]. Menstrual disorders were reported based on the last 12 months [33,34,35,36,37,67,68,71,74], the last 3 months [30], one year after menarche [75], or in the post-menarche period [70]. The highest prevalence of primary amenorrhea (53.8%), secondary amenorrhea (50%) and oligomenorrhea (61%) was observed in rhythmic gymnastics [31,35,36]. The highest prevalence of MC irregularities (55%) and hypermenorrhea (62.22%) was also observed in rhythmic gymnastics [67,71].

#### 3.2.4. Winter and Outdoor Sports

Only one study focused on the prevalence of menstrual disorders in winter sports during the previous 12 months. This study did report the prevalence of amenorrhea and oligomenorrhea in different sports modalities. Secondary amenorrhea and oligomenorrhea were prevalent in 22.5% and 30% of winter sports athletes [76], respectively (Table 5).

**Figure 2:**
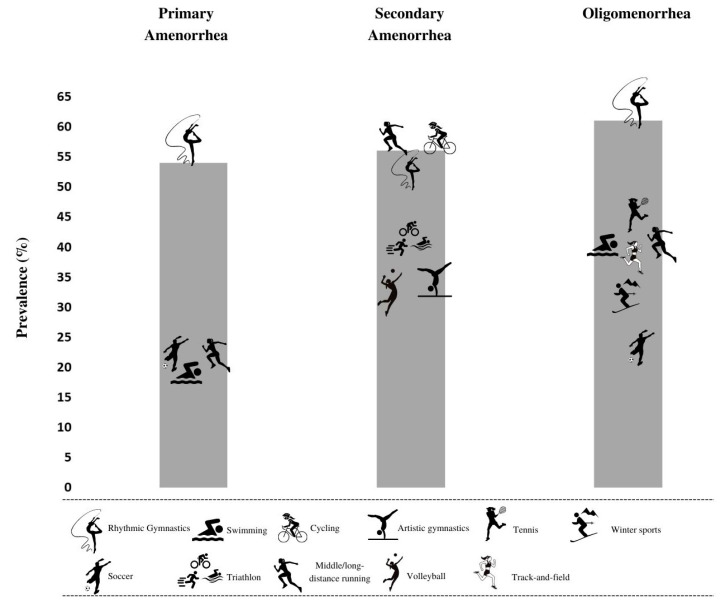
Sports disciplines with the highest prevalence of primary, secondary amenorrhea and oligomenorrhea.

In Table 6 the mean prevalence of menstrual cycle disorders and the minimum and maximum prevalence when more than one study was available in analyzed sports disciplines is summarized. These pooled data show that the highest mean prevalence of primary amenorrhea can be found in rhythmic gymnastics (25%), soccer (20%) and swimming (19%). The highest mean prevalence of secondary amenorrhea was observed in cycling (56%), triathlon (40%) and rhythmic gymnastics (31%). The highest mean prevalence of oligomenorrhea was observed in boxing (55%), rhythmic gymnastics (44%) and artistic gymnastics (32%). No menstrual disorders were found among athletes competing in water polo. Figure 2 shows the highest prevalence of primary, secondary amenorrhea and oligomenorrhea considering the individual results from the articles included in the study.

## 4. Discussion

The major findings of the present rapid review were that rhythmic gymnastics is a discipline in which the athletes suffer a major risk to present menstrual disorders, including a percentage of 53.8% for primary amenorrhea [35], 30.8% for secondary amenorrhea [36] and 61% for oligomenorrhea [31]. Moreover, cyclic and individual sports disciplines also presented high frequencies of menstrual disorders, especially middle/long distance running (55% for secondary amenorrhea [39]), cycling (55.6% for secondary amenorrhea [46]), triathlon (40% for secondary amenorrhea [64]), boxing (54.6% for oligomenorrhea [74]) and tennis (42.9% for oligomenorrhea [33]). In team sports, a higher percentage of menstrual disorders was found in volleyball athletes, showing a prevalence of secondary amenorrhea of 30% amongst the athletes [39]). Lastly, secondary amenorrhea and oligomenorrhea were menstrual disorders with major prevalence in the sports disciplines.

### 4.1. Primary Amenorrhea

As described previously, primary amenorrhea can be known as a failure to reach the first menstrual period. In the general population, the occurrence of this event is less than 1% [6]; nonetheless, in the athletes’ population, it was possible to notice that this prevalence is considerably higher, reaching 53.8% as demonstrated in rhythmic gymnastics athletes [35]. Besides the higher prevalence in rhythmic gymnastics, primary amenorrhea was also frequent in soccer players (20%) [33] and in swimmers (19%) [33].

A delay in menarche in athletes compared to controls has been demonstrated in the scientific literature [39,77,78]. Not just elite but novice athletes also showed a significant delay of around half a year [78]. A possible explanation could be related to the training intensity combined with inadequate recovery. It can result in an energy deficit leading to suppression of the endocrine function and hypoestrogenemia, causing the delay of age of menarche [79,80,81].

Previous studies show that in elite rhythmic gymnasts, intensive training and negative energy balance delays the pubertal onset by affecting the HPO axis and decreasing the estrogen production; thus, the pre-pubertal stage is prolonged and the pubertal development occurs in later age [80,81]. On the other hand, it has been described that swimmers present a greater percentage of subcutaneous fat compared to other athletes as it reflects the adjustment to the sport-specific requirements. As so, the delayed puberty in swimmers has been associated with mild hyperandrogenism, an androgen disorder characterized by hirsutism, acne or alopecia, and mild insulin resistance presenting a metabolic and cardiovascular risk [82], as reported somewhere [80].

### 4.2. Secondary Amenorrhea

Secondary amenorrhea was one of the menstrual disorders that presented the highest prevalence in sports disciplines such as cycling (56% [46]), triathlon (40% [64]) and rhythmic gymnastics (mean prevalence of 31%). The prevalence reported in the studies can be considered elevated compared to the general population in which the estimated prevalence is around 5–12.2% [83,84].

Conceptualized as the absence of menstruation for three or more months in women with previous regular menses, or for 6 months in women with previously irregular menses [7,8], secondary amenorrhea in athletes is likely related to the deficit of energy as suggested in the studies that evaluated the cyclists, triathletes and rhythmic gymnasts [36,46,64]. Sixty percent of the triathletes were found to be in a caloric deficit [64]. Similarly, in the study with elite cyclists, an increased risk of energy deficiency was reported, as their current body weight was not considered ideal for performance in races stressing out the lean body image [46]. Similarly, in rhythmic gymnastics, the lean-sport discipline, intensive training and the pressure for a lean body can result in low body weight and diet restrictions and consequently an inadequate energy availability [36].

Athletes with secondary amenorrhea also can acquire a poor or reduced bone mineral density and a high incidence of bone fractures [85] as the consequence of hypoestrogenemia and chronically low energy availability [2]. In the long-term, hypoestrogenemia is associated with cardiovascular risk, increasing the risk of atherosclerosis and bone loss [80].

### 4.3. Oligomenorrhea

Oligomenorrhea corresponds to prolonged menstrual periods (e.g., 35 days or more) or irregular cycles (e.g., 5 to 7 cycles per year) [14,15]. According to previous studies, the prevalence of oligomenorrhea in the general population ranges from 6–15.3% [15,16,86,87]. In our review, the highest mean prevalence of oligomenorrhea was observed in boxing (55%), rhythmic gymnastics (44%) and artistic gymnastics (32%). Energy imbalance is again highlighted as the main reason for the presence of oligomenorrhea i The authors speculate that the lower body fat mass and a great energy expenditure in training could generate a lower energy availability triggering the occurrence of oligomenorrhea amongst the athletes from boxing and gymnastics disciplines [36,73,74]. Non-pharmacological treatment focusing on the resumption of menses should be prioritized in athletes, as the menses and normal estrogen status are of great importance for bone health [88].

Taken together, the menstrual disorders mentioned above presented a great influence on low energy availability, as reported in the studies included. Therefore, beyond the prevalence of menstrual disorders in sports disciplines, coaches, trainers and physicians should also be aware of the concept of low energy availability. Dietary and training interventions in female athletes engaged not only in aesthetic disciplines such as rhythmic gymnastics, but also in cycling, winter sports or team sports disciplines (e.g., [33,39,40,76]) are vital.

### 4.4. Methodological Aspects

Additional to the outcomes from the prevalence of menstrual disorders, this review also shows that the definition of menstrual disorders and the methods of evaluation varied among the studies. There is a need for clear guidelines on how to design studies about the prevalence and how to report the outcomes as mentioned in a previous prevalence review [89].

The menstrual disorders evaluated were conditioned to the methodology chosen by each study. A retrospective report ranged from the last 5 years [41], 12 months (e.g., [33,34,42]), 6 months (e.g., [43]), 3 months [30], one year after menarche [75], or in post-menarche (e.g., [39,44,45]). Similarly, different definitions of menstrual disorders were used amongst the studies. In some, the definition of primary amenorrhea was the non-appearance of menarche up to 15 years of age (e.g., [37,40,50]) and for others the non-appearance up to 16 years of age (e.g., [33,39,43]). The definition of secondary amenorrhea differed from not having a menstrual period for 6 months [48], the absence of three consecutive menstrual cycles [39,50], or having 0 to 3 periods per year [34,44]. The definition of oligomenorrhea included having 4 to 9 periods per year [34], having 6 to 9 periods per year [33], or an interval between periods of more than 35 days and less than 90 days [35,49] (see Supplementary Materials Table S3).

The comparison between different sports disciplines using the same menstrual disorder concept and study methodology was reported by three studies [33,34,39]. In the study of Dusek [39], a highest prevalence of primary amenorrhea and secondary amenorrhea were observed in long-distance runners (20% and 55%, respectively) compared to short distance runners, basketball and volleyball players. Mudd et al. [34] observed the highest prevalence of secondary amenorrhea in short distance runners (25%) and oligomenorrhea in long-distance runners (28%) compared to gymnasts, field hockey players, soccer players, rowers and swimmers. Tenforde et al. [33] reported the highest prevalence of primary amenorrhea in gymnasts (37.6%), secondary amenorrhea in swimmers (23.8%) and oligomenorrhea in tennis players (42%) compared to basketball players, cross-country runners, rowers, soccer players, field-hockey players, fencers, volleyball and water polo players. Further research should monitor the occurrence of menstrual cycle disorders of athletes from different sports disciplines in association with their energy availability to provide a more detailed insight into this topic.

### 4.5. Limitations of the Study

The main limitation related to the rapid review is the use of two databases to perform the search, so some studies could be missing. Outcomes from only one sport discipline was used, for example in the winter sports, boxing, fencing, figure skating, water polo, or ice hockey, which should be taken into consideration on generalization on the results from these sport disciplines. Differences in sample sizes, measurement methodology, menstrual disorders definition and monitored period, as well as including studies with fair quality requires caution when interpretating the results of this review.

## 5. Conclusions

In conclusion, the prevalence of menstrual disorders amongst the athletes ranged from non-disorder (0%) to a maximum percentage of 61%. The systematically summarized studies support the notion of a higher prevalence of menstrual disorders in sports as gymnastics and endurance disciplines. However, team-sports modalities such as volleyball and soccer also presented a considerable percentage of menstrual disorders compared to the general population. As a practical implication, the review reinforces the importance of coaches and physicians, especially from the sports disciplines with a high risk of menstrual disorders, to monitor the menstrual cycle regularity of the athletes as the occurrence of these disorders can be associated with impairment on some health components.

## Figures and Tables

**Table 1:** Sports disciplines and the number of studies assessing the prevalence of menstrual cycle disorders.

Team Sports	*n*	Cyclic Sports	*n*	Other Individual Sports	*n*	Winter and Outdoor Sports	*n*
Basketball	2	Cross-country running	6	Boxing	1	Alpine skiing	1
Field hockey	2	Middle/long-distance running	10	Fencing	1	Biathlon	1
Ice hockey	1	Rowing	4	Figure skating	1	Bobsleigh	1
Soccer	3	Short distance running	2	Gymnastics *	2	Luge	1
Softball	2	Swimming	4	Artistic gymnastics	2	Skeleton	1
Synchronized swimming	2	Track-and-field	6	Rhythmic gymnastics	10	Snowboarding	1
Volleyball	2	Triathlon	3	Tennis	2	Aerial skiing	1
Water polo	1	Cycling	2			Speed skating	1

* Not specified if artistic or rhythmic gymnasts participated in the study [33,34].

**Table 2:** Sample characteristics, menstrual disorders measurement and prevalence in team sports.

Study (Year)	Sport	Sample (*n*)	Mean Age ± SD (Years)	Methods	Monitored Period	Age of Menarche (Years)	Primary Amenorrhea (*n*, %)	Secondary Amenorrhea (*n*, %)	Oligomenorrhea (*n*, %)	Other (*n*, %)
Dusek (2001) [39]	basketball	18	18.8 ± 1.1	questionnaire	post-menarche	12.7 ± 1.1	0 (0.0%)	1 (5.6%)	NA	painful menstruation 18 out of 67 athletes (26.9%)
Tenforde et al. (2017) [33]	basketball	9	20 ± 1.3	PPEs	12 months	NA	0 (0.0%)	0 (0.0%)	0 (0.0%)	NA
Tenforde et al. (2017) [33]	field hockey	21	20 ± 1.3	PPEs	12 months	NA	2 (9.55%)	3 (14.3%)	4 (19.1%)	NA
Mudd, Fornetti and Pivarnik (2007) [34]	field hockey	10	19.8 ± 1.2	questionnaire	12 months	12.9 ± 1.6	NA	1 (10.0%)	0 (0.0%)	NA
Egan et al. (2003) [37]	ice hockey	28	23.5 ± 4.8	questionnaire	12 months	13.3 ± 1.3	4 (15.0%)	NA	2 (7.1%)	71.5% experienced minor menstrual dysfunction
Prather et al. (2016) [40]	soccer	145	16.4 ± 4	questionnaire	1 year post-menarche	13.0 ± 1.0	28 (19.3%) *		NA	NA
Tenforde et al. (2017) [33]	soccer	5	20 ± 1.3	PPEs	12 months	NA	1 (20.0%)	0 (0.0%)	1 (20.0%)	NA
Mudd, Fornetti and Pivarnik (2007) [34]	soccer	10	19.8 ± 0.9	questionnaire	12 months	12.9 ± 1.4	NA	1 (10.0%)	1 (10.0%)	NA
Tenforde et al. (2017) [33]	softball	19	20 ± 1.3	PPEs	12 months	NA	3 (15.75%)	2 (10.5%)	2 (10.5%)	NA
Mudd, Fornetti and Pivarnik (2007) [34]	softball	14	20.1 ± 1.1	questionnaire	12 months	13.5 ± 2.5	NA	1 (7.1%)	1 (7.1%)	NA
Ramsay and Wolman (2001) [38]	synchronized swimming	23	17.1 ± 1.9	questionnaire	6 months	NA	NA	0 (0.0%)	3 (13.0%)	NA
Tenforde et al. (2017) [33]	synchronized swimming	11	20 ± 1.3	PPEs	12 months	NA	1 (9.1%)	1 (9.1%)	2 (18.2%)	NA
Dusek (2001) [39]	volleyball	10	17.4 ± 1.4	questionnaire	post-menarche	13.3 ± 1.5	0 (0.0%)	3 (30.0%)	NA	NA
Tenforde et al. (2017) [33]	volleyball	9	20 ± 1.3	PPEs	12 months	NA	0 (0.0%)	1 (11.1%)	1 (11.1%)	NA
Tenforde et al. (2017) [33]	water polo	16	20 ± 1.3	PPEs	12 months	NA	0 (0.0%)	0 (0.0%)	0 (0.0%)	NA

PPEs: Preparticipation Physical Examinations; * reported prevalence of primary and/or secondary amenorrhea.

**Table 3:** Sample characteristics, menstrual disorders measurement and prevalence in cyclic sports.

Study (Year)	Sport	Sample (*n*)	Mean Age ± SD (Years)	Methods	Monitored Period	Age of Menarche (Years)	Primary Amenorrhea (*n*, %)	Secondary Amenorrhea (*n*, %)	Oligomenorrhea (*n*, %)	Other (*n*, %)
Thompson (2007) [48]	cross-country running	300	19.64 ± 1.56	questionnaire	post menarche period	13.46 ± 1.65	NA	16 (5.3%)	53 (17.7%)	NA
Jesus et al. (2021) [42]	cross-country running	83	21.8 ± 4.0	LEAF-Q	12 months	NA	NA	18 (22.0%)	23 (28.0%)	NA
Barrack, Rauch, and Nichols (2008) [49]	cross-country running	93	16.1 ± 0.1	questionnaire. preparticipation medical history form	12 months	12.8 ± 0.1	3 (3.2%)	16 (17.2%)	5 (5.4%)	NA
Barrack et al. (2010) [50]	cross-country running	39	15.7 ± 0.2	questionnaire	12 months	13.9	3 (7.7%)	9 (23.1%)	9 (23.1%)	NA
Mudd, Fornetti and Pivarnik (2007) [34]	cross-country and track runners (800 m or longer)	25	20.4 ± 1.3	questionnaire	12 months	13.8 ± 2.1	NA	4 (16.0%)	7 (28.0%)	MC irregularity: 25 (25.2%)
Tenforde et al. (2017) [33]	cross-country running	47	20 ± 1.3	PPEs	12 months	NA	9 (19.17%)	10 (21.3%)	8 (17.0%)	NA
Hutson et al. (2021) [51]	middle/long-distance running	183	18–40	questionnaire	12 months	NA	NA	1 (0.5%)	37 (20.2%)	NA
Muia et al. (2015) [52]	middle/long-distance running	56	16	questionnaire	12 months	14	24 (13.1%)	36 (19.7%)	NA	NA
Beckvid Henriksson, Schnell, and Lindén Hirschberg (2000) [53]	middle/long-distance running	93	25.8 ± 1.3	questionnaire	12 months	14.2 ± 0.5	1 (1.1%)	7 (8.0%)	16 (17.0%)	NA
Pollock et al. (2010) [32]	middle/long-distance running	36	22.9 ± 6.0	questionnaire	12 months	13.9 ± 1.5	NA	9 (25.0%)	14 (38.0%)	NA
Burrows et al. (2003) [54]	middle/long-distance running	52	31.0 ± 5.0	questionnaire	12 months	14 ± 2	NA	1 (2.0%)	11 (21.2%)	NA
Cobb et al. (2003) [55]	middle/long-distance running	91	21.8 ± 0.5	questionnaire	12 months	13.8 ± 0.2	NA	9 (10.0%)	24 (26.0%)	NA
Dusek (2001) [39]	middle/long-distance running	20	17.9 ± 2.1	questionnaire	post-menarche	13.5 ± 1.7	4 (20.0%)	11 (55.0%)	NA	NA
Tenforde et al. (2015) [56]	middle/long-distance running	91	16.9 ± 1.3	PPEs	12 months	12.9 ± 1.5	39 (43.0%) *		NA	NA
Rauh, Barrack and Nichols (2014) [57]	middle/long-distance running	89	15.5 ± 1.3	questionnaire	12 months	12.3 ± 1.1	NA	NA	NA	MC irregularity: 19 (21.3%)
Gibson et al. (2000) [44]	middle/long-distance running	51	16–35	questionnaire	Post-menarche period	NA	NA	NA	NA	MC irregularity: 33 (64.7%)
Walsh, Crowell and Merenstein (2020) [58]	lightweight rowers	78	20 ± 2.8	questionnaire	post-menarche period	NA	NA	NA	NA	MC irregularity: 14 (35%)
	openweight rowers	80	21.5 ± 7			NA	NA	NA	NA	MC irregularity: 8 (22.9%).
Dimitriou et al. (2014) [45]	lightweight rowers	12	26.6 ± 2.0	questionnaire	post-menarche period	13.4 ± 1.1	NA	NA	NA	MC irregularity: 10 (83.3%)
Tenforde et al. (2017) [33]	rowing/crew	30	20 ± 1.3	PPEs	12 months	NA	2 (6.6%)	1 (3.3%)	5 (16.7%)	NA
Mudd, Fornetti and Pivarnik (2007) [34]	rowing/crew	15	20.5 ± 2.1	questionnaire	12 months	13.2 ± 2.4	NA	0 (0.0%)	2 (13.3%)	NA
Mudd, Fornetti and Pivarnik (2007) [34]	short-distance running	8	20.1 ± 1.3	questionnaire	12 months	13.4 ± 1.9	NA	2 (25.0%)	0 (0.0%)	NA
Dusek (2001) [39]	short-distance running	14	17.9 ± 2.1	questionnaire	post-menarche	13.1 ± 0.8	0 (0.0%)	3 (21.4%)	NA	NA
Coste et al. (2011) [59]	swimming	18	15.2 ± 1.1	questionnaire	post-menarche	12.5 ± 1.0	NA	2 (11.1%)	7 (38.9%)	NA
Schtscherby et al. (2009) [43]	swimming	78	16.7 ± 1.22	questionnaire	6 months	12.38 ± 0.2	NA	0 (0.0%)	15 (19.2%)	NA
Mudd, Fornetti and Pivarnik (2007) [34]	swimming/diving	9	20.4 ± 1.1	questionnaire	12 months	13.5 ± 1.5	NA	0 (0.0%)	2 (22.2%)	NA
Tenforde et al. (2017) [33]	swimming/diving	21	20 ± 1.3	PPEs	12 months	NA	4 (19.0%)	5 (23.8%)	2 (9.5%)	NA
Tsukahara et al. (2021) [60]	track-and-field	91	18.10 ± 0.37	interview	12 months	13.4 ± 1.3	NA	6 (6.6%)	33 (36.3%)	NA
Sygo et al. (2018) [61]	track-and-field	13	21 ± 3	LEAFNAQ	12 months	NA	NA	NA	NA	MC irregularity: 3 (23%)
Robbeson, Havemann-Nel and Wright (2013) [62]	track-and-field	16	19.0	questionnaire	12 months	14	NA	3 (18.75%)	1 (6.25%)	NA
Nattiv et al. (2013) [41]	track-and-field	22	20.2 ± 0.3	questionnaire	5-year period	NA	NA	NA	NA	MC irregularity: 5 (23%)
Tenforde et al. (2017) [33]	track-and-field	4	20 ± 1.3	PPEs	12 months	NA	0 (0.0%)	0 (0.0%)	1 (25.0%)	NA
Feldmann et al. (2011) [63]	track-and-field	103	16 ± 0.97	questionnaire	12 months	NA	NA	16 (16.7%)	16 (16.7%)	NA
Hoch, Stavrakos and Schimke (2007) [64]	triathletes	15	35 ± 6	questionnaire	post-menarche	NA	NA	6 (40.0%)	NA	NA
Duckham et al. (2015) [47]	endurance athletes (running, triathlon)	61	25.3 ± 7.3	questionnaire	12 months	14.0 ± 0.2	NA	NA	29 (47.5%)	NA
Duckham et al. (2013) [65]	endurance athletes (runners, triathletes)	68	25.4	questionnaire	12 months	14.2	NA	NA	24 (35.0%)	NA
Raymond-Barker, Petroczi and Quested (2007) [66]	endurance athletes (runners, cyclists), gymnasts	48	37 ± 7.95	questionnaire	post-menarche period	NA	NA	14 (23.7%)	NA	NA
Haakonssen et al. (2014) [46]	cyclists	37	18–36	Female Cyclist Weight Management Questionnaire (FCWM)	12 months	NA	NA	10/18 (55.6%)	1 (5.6%)	NA

PPEs: Preparticipation Physical Examinations; * reported prevalence of primary and/or secondary amenorrhea.

**Table 4:** Sample characteristics, menstrual disorders measurement and prevalence in other individual sports.

Study (Year)	Sport	Sample (*n*)	Mean Age ± SD (Years)	Methods	Monitored Period	Age of Menarche (Years)	Primary Amenorrhea (*n*, %)	Secondary Amenorrhea (*n*, %)	Oligomenorrhea (*n*, %)	Other (*n*, %)
Trutschnigg et al. (2008) [74]	boxing	11	26.7 ± 6.8	modified ACSM medical health questionnaire	12 months	12.8 ± 1.9	NA	0 (0.0%)	6 (54.6%)	NA
Tenforde et al. (2017) [33]	fencing	5	20 ± 1.3	PPEs	12 months	NA	0 (0.0%)	0 (0.0%)	1 (20.0%)	NA
Egan et al. (2003) [37]	figure skating	37	17.5 ± 3.4	questionnaire	12 months	NA	NA	3 (10.56%)	8 (28.15%)	NA
Tenforde et al. (2017) [33]	gymnastics	16	20 ± 1.3	PPEs	12 months	NA	6 (37.6)	3 (18.8%)	3 (18.8%)	NA
Mudd, Fornetti and Pivarnik (2007) [34]	gymnastics	8	19.7 ± 0.9	questionnaire	12 months	14.3 ± 1.3	NA	1 (12.5%)	2 (25.0%)	NA
Corujeira et al. (2012) [73]	artistic gymnasts	27	14.08	questionnaire	NA	13	0 (0.0%)	4 (14.0%)	8 (29.0%)	NA
Helge and Kanstrup (2002) [72]	artistic gymnasts	6	17.9 ± 1.5	questionnaire	NA	15.3 ± 1.8	1 (16.65%)	2 (33.3%)	2 (33.3%)	NA
Meng et al. (2020) [35]	rhythmic gymnastics	52	20 ± 3.0	LEAF-Q	12 months	NA	28 (53.8%)	16 (30.8%)	7 (13.5%)	NA
Roupas et al. (2014) [68]	rhythmic gymnastics	77	18.3 ± 2.6	questionnaire	12 months	NA	18 (23.4%)	NA	35 (45.5%)	NA
Maїmoun et al. (2013) [67]	rhythmic gymnastics	82	18.3 ± 2.5	questionnaire	12 months	15.6 ± 1.6	17 (20.7%)	NA	NA	MC irregularity: 36 (55%)
Salbach et al. (2007) [69]	rhythmic gymnastics	50	14.8 ± 2.1	questionnaire	NA	NA	7 (14.0%) *		NA	NA
Muñoz et al. (2004) [70]	rhythmic gymnastics	9	16.2 ± 2.0	individual medical examination	post-menarche period	15 ± 1	NA	NA	4 (45.0%)	NA
Czajkowska et al. (2019) [71]	rhythmic gymnastics	45	16.28 ± 0.84	questionnaire	12 months	13.02 ± 1.03	NA	8 (47.06%)	9 (52.94%)	hypermenorrhoea 28 (62.22%)
Klinkowski et al. (2008) [30]	rhythmic gymnastics	51	15.2 ± 1.8	questionnaire	3 months	NA	0 (0.0%)	4 (7.8%)	NA	NA
Klentrou and Plyley (2003) [31]	rhythmic gymnastics	23	14.7 ± 0.4	survey	post-menarche period	13.6 ± 1.2	NA	4 (17.4%)	14 (61.0%)	NA
Di Cagno et al. (2012) [36]	rhythmic gymnastics	46	17.4 ± 3.0	menstrual history questionnaire (MHQ)	12 months	15.0 ± 1.5	NA	23 (50.0%)	NA	NA
Helge and Kanstrup (2002) [72]	rhythmic gymnastics	5	18.3 ± 1.9	questionnaire	NA	13.3 ± 1.8	0 (0.0%)	0 (0.0%)	1 (20.0%)	NA
Tenforde et al. (2017) [33]	tennis	7	20 ± 1.3	PPEs	12 months	NA	0 (0.0%)	1 (14.3%)	3 (42.9%)	NA
Coelho et al. (2013) [75]	tennis	24	14.77 ± 2.16	questionnaire	1 year after menarche	12.05 ± 1.25	NA	2 (9.1%)	1 (4.5%)	MC irregularity: 8 (33.3%)

PPEs: Preparticipation Physical Examinations; * reported prevalence of primary and/or secondary amenorrhea.

**Table 5:** Sample characteristics, menstrual disorders measurement and prevalence in winter and outdoor sports.

Study (Year)	Sport	Sample (*n*)	Mean Age ± SD (Years)	Methods	Monitored Period	Age of Menarche	Primary Amenorrhea	Secondary Amenorrhea	Oligomenorrhea (*n*, %)	Other (*n*, %)
Meyer et al. (2004) [76]	long track speed skating, freestyle skiing, aerials, snowboarding, alpine skiing, biathlon, bobsleigh, skeleton, luge	*n* = 40	26.1 ± 5.7	questionnaire	12 months	13.4 ± 1.5	NA	*n* = 9 (22.5%)	*n* = 12 (30.0%)	NA

**Table 6:** Sports disciplines and the mean (min, max) prevalence (%) of menstrual cycle disorders.

	Primary Amenorrhea	Secondary Amenorrhea	Oligomenorrhea
Team Sports			
Basketball	0.00%	2.80% (0.00, 5.60)	0.00%
Field hockey	9.55%	12.15% (10.00, 14.30)	9.55% (0.00, 19.10)
Ice hockey	15.00%	NA	7.10%
Soccer	20.00%	5.00% (0.00, 10.00)	15.00% (10.00, 20.00)
Softball	15.75%	8.80% (7.10, 10.50)	8.80% (7.10, 10.50)
Synchronized swimming	9.10%	4.55% (0.00, 9.1)	15.60% (13.00, 18.20)
Volleyball	0.00%	20.55% (11.10, 30.00)	11.10%
Water polo	0.00%	0.00%	0.00%
Cyclic sports			
Cross-country running	10.02% (3.20, 19.17)	17.48% (5.30, 23.10)	19.87% (5.40, 28.00)
Middle/long-distance running	11.40% (1.10, 20.00)	17.17% (0.50, 55.00)	24.48% (17.00, 38.00)
Rowing	6.60%	1.65% (0.00, 3.30)	15.00% (13.30, 16.70)
Short distance running	0.00%	23.20% (21.4, 25.00)	0.00%
Swimming	19.00%	8.73% (0.00, 23.80)	22.45% (9.50, 38.90)
Track-and-field	0.00%	10.51% (0.00, 18.75)	21.04% (6.25, 36.30)
Triathlon	NA	40.00%	NA
Cycling	NA	55.60%	5.60%
Other Individual Sports			
Boxing	NA	0.00%	54.60%
Fencing	0.00%	0.00%	20.00%
Figure skating	NA	10.56%	28.15%
Artistic gymnastics	8.33% (0.00, 16.65)	23.67% (14.00, 33.33)	31.17% (29.00, 33.33)
Rhythmic gymnastics	24.48% (0.00, 53.80)	30.61% (7.80, 50.00)	43.59% (13.50, 61.10)
Tennis	0.00%	11.70% (9.10, 14.30)	23.70% (4.50, 42.90)
Winter sports	NA	22.50%	30.00%

## Data Availability

No data was created in this study. Data sharing is no applicable to this article.

## Supplementary Materials

The following supporting information can be downloaded at: https://www.mdpi.com/article/10.3390/ijerph192114243/s1, Table S1: Checklist for quality assessment of the studies; Table S2: Adjusted Downs and Black Assessment Checklist evaluation; Table S3: Supplementary data.
