# Supplementary material for: The Prevalence of Menstrual Cycle Disorders in Female Athletes from Different Sports Disciplines: A Rapid Review
Source: Int J Environ Res Public Health. 2022 Oct 31;19(21):14243. doi: 10.3390/ijerph192114243 (PMC9658102; doi:10.3390/ijerph192114243)
Supplement: Supplementary file 1 [file ijerph-19-14243-s001.zip › ijerph-1897784-supplementary.pdf]

Table S1: Checklist for quality assessment of the studies.

Adjusted Downs and Black Quality Assessment Checklist (1998) [24]		
**Item**	**Criteria**	**Yes (1),<br>No/<br>Unable to determine (0)**
**Reporting**		
1.	Is the hypothesis/aim/objective clearly described?	
2.	Are the main outcomes to be measured clearly described in the Introduction or Methods section?	
3.	Are the characteristics of the participants included in the study clearly described?	
6.	Are the main findings of the study clearly described?	
7.	Does the study provide estimates of the random variability for the main outcomes?	
10.	Have the probability values been reported for the main outcomes?	
**External validity**		
11.	Were the subjects asked to participate in the study representative of the entire population from which they were recruited?	
12.	Were those subjects who were prepared to participate representative of the entire population from which they were recruited?	
**Internal validity – bias**		
16.	If any of the results of the study were based on "data dredging", was this made clear?	
**Item**	**Criteria**	**Yes (1),<br>No/<br>Unable to determine (0)**
18.	Were the statistical tests used to assess the main outcomes appropriate?	
20.	Were the menstrual cycle regularity measures accurate (valid and reliable)?	
21.	Were the participants in different intervention groups (trials and cohort studies) or were the cases and controls (case-control studies) recruited from the same population?	
22.	Were study participants in different intervention groups (trials and cohort studies) or were the cases and controls (case-control studies) recruited over the same period of time?	

Table S2. Adjusted Downs and Black Assessment Checklist evaluation.

Study	1	2	3	6	7	10	11	12	16	18	20	21	22	Total score	Kennelly score (%)
Tsukahara et al. (2021) [60]	1	1	1	1	1	1	1	1	1	1	0	1	1	12	92,3
Hutson et al. (2021) [51]	1	1	1	1	1	1	0	1	1	1	1	1	1	12	92,3
Tenforde et al. (2015) [56]	1	1	1	1	1	1	0	1	1	1	1	1	0	11	84,6
Feldmann et al., (2011) [63]	1	1	1	1	1	1	0	0	1	1	1	1	1	11	84,6
Coelho et al. (2013) [75]	1	1	1	1	1	1	0	0	1	1	1	1	1	11	84,6
Coste et al. (2011) [59]	1	1	1	1	1	1	0	0	1	1	1	1	1	11	84,6
Schtscherbyna et al. (2009) [43]	1	1	1	1	1	0	0	1	1	1	1	1	1	11	84,6
Jesus et al. (2021) [42]	1	1	0	1	1	1	0	1	1	1	1	1	1	11	84,6
Gibson et al. (2000) [44]	1	1	1	1	1	1	0	0	1	1	1	1	1	11	84,6
Meng et al. (2020) [35]	1	1	1	1	1	1	0	0	1	1	1	1	0	10	76,9
Haakonssen et al. (2014) [46]	1	1	0	1	1	1	0	0	1	1	1	1	1	10	76,9
Rauh et al. (2014) [57]	1	1	0	1	1	1	0	0	1	1	1	1	1	10	76,9

Roupas et al. (2014) [68]	1	1	0	1	1	1	0	0	1	1	1	1	1	10	76,9
Robbeson et al. (2012) [62]	1	1	1	1	1	1	0	0	1	1	1	1	0	10	76,9
Corujeira et al. (2012) [73]	1	1	0	1	1	1	0	1	1	1	0	1	1	10	76,9
Barrack et al. (2010) [50]	1	1	1	1	1	0	0	0	1	1	1	1	1	10	76,9
Barrack et al. (2008) [49]	1	1	1	1	1	0	0	0	1	1	1	1	1	10	76,9
Klinkowski et al. (2008) [30]	1	1	1	1	1	1	0	0	1	1	1	1	0	10	76,9
Salbach et al. (2007) [69]	1	1	1	1	1	1	0	1	1	0	0	1	1	10	76,9
Burrows et al. (2003) [54]	1	1	1	1	1	1	0	0	1	1	1	1	0	10	76,9
Raymond-Barker et al. (2007) [66]	1	1	1	1	1	1	0	1	1	1	1	0	0	10	76,9
Sygo et al. (2018) [61]	1	1	1	0	1	1	0	0	1	1	0	1	1	9	69,2
Walsh et al. (2020) [58]	1	1	0	1	1	1	0	0	1	1	1	1	0	9	69,2
Maïmoun et al. (2013) [67]	1	1	0	1	1	0	0	0	1	1	1	1	1	9	69,2
Duckham et al. (2013) [65]	1	1	1	1	1	1	0	0	1	1	1	0	0	9	69,2
Pollock et al. (2010) [32]	1	1	0	1	1	1	0	0	1	1	1	1	0	9	69,2

Trutschnigg et al. (2008) [74]	1	1	1	1	1	0	0	0	1	1	1	1	0	9	69,2
Thompson (2009) [48]	1	1	0	1	1	1	0	0	1	1	1	1	0	9	69,2
Mudd et al. (2007) [34]	1	1	1	1	1	0	0	1	1	1	1	0	0	9	69,2
Meyer et al. (2004) [76]	1	1	1	1	1	1	0	0	1	1	1	0	0	9	69,2
Cobb et al. (2003) [55]	1	1	1	1	1	0	0	0	1	1	1	1	0	9	69,2
Helge and Kanstrup (2002) [72]	1	1	1	1	1	0	0	0	1	1	0	1	1	9	69,2
Beckvid Henriksson et al. (2000) [53]	1	1	0	1	1	0	0	0	1	1	1	1	1	9	69,2
Dimitriou et al. (2013) [45]	1	1	1	1	1	0	0	0	1	1	1	1	0	9	69,2
Nattiv et al. (2013) [41]	1	1	0	1	1	1	0	0	1	1	0	1	1	9	69,2
Czajkowska et al. (2013) [71]	1	1	0	1	1	0	0	0	1	1	1	1	0	8	61,5
Tenforde et al. (2017) [33]	1	1	0	1	1	1	0	0	1	1	1	0	0	8	61,5
Prather et al. (2016) [40]	1	0	0	0	1	1	0	0	1	1	1	1	1	8	61,5
Muia et al. (2015) [52]	0	0	1	1	1	1	0	0	1	1	1	1	0	8	61,5

Di Cagno et al. (2012) [36]	1	1	0	0	1	0	0	0	1	1	1	1	1	8	61,5
Dusek (2001) [39]	1	1	0	1	1	1	0	0	1	1	1	0	0	8	61,5
Duckham et al. (2015) [47]	0	1	1	0	1	0	0	0	1	1	1	0	1	7	53,8
Hoch et al. (2007) [64]	1	1	1	1	1	0	0	0	1	0	0	1	0	7	53,8
Muñoz et al. (2004) [70]	1	1	0	1	1	0	0	0	1	1	0	1	0	7	53,8
Klentrou and Plyley (2003) [31]	1	1	0	1	1	0	0	0	1	1	0	1	0	7	53,8
Egan et al. (2003) [37]	0	1	0	1	1	0	0	0	1	1	1	0	1	7	53,8
Ramsay and Wolman (2001) [38]	1	1	0	0	1	0	0	0	1	0	1	1	1	7	53,8

Table S3: Supplementary data.

Study	Country	Body mass (kg)	Body height (m)	Trainings (hours per week)	Primary amenorrhea definition	Secondary amenorrhea definition	Oligomenorrhea definition
**Team sports**							
Dusek (2001) [39]	Croatia	NA	NA	18 ± 0	non-appearance of menarche up to 16 years of age	absence of menstruation for more than 3 months in the post-menarche period	NA
Tenforde et al. (2017) [33]	USA	NA	NA	NA	non-appearance of menarche up to 16 years of age	less than 6 periods per year	6-9 periods per year
Mudd, Fornetti and Pivarnik (2007) [34]	USA	63.2 ± 10.6	1.66 ± 0.08	NA (collegiate athletic association division I university)	NA	0 to 3 menstrual cycles per year	4-9 cycles per year
Egan et al. (2003) [37]	Sweden, USA, Canada national teams	68.2 ± 1.2	1.68 ± 0.01	8.1 ± 1.8 sessions/week	non-appearance of menarche up to 15 years of age	three or less menstruations per year	cycles of between 45 and 90 days
Prather et al. (2016) [40]	USA	NA	NA	9,5 ± 6	non-appearance of menarche up to 15 years of age	3 or more missed periods in the past year	NA
Ramsay and Wolman (2001) [38]	UK	55,7 ± 6,9	165 ± 7	NA	NA	no more than one period in the six months	cycle length of greater than 35 days
**Cyclic sports**							

Thompson (2007) [48]	USA	NA	NA	9,1 ± 5,7	NA	have not had a menstrual period for 6 months	have a menstrual period every 6 weeks
Jesus et al. (2021) [42]	Portugal	51,0 ± 4,1	165,8 ± 5,1	14,8 ± 5,4	NA	cycles 4 and more months long	cycles 2-3 months long
Barrack, Rauch, and Nichols (2008) [49]	USA	55,9 ± 0,7	164,8 ± 0,7	NA	no onset of menarche by age 15 years	absence of three consecutive menstrual cycles in the past year	interval between menses more 35 days and less than 90 day in the past year
Barrack et al. (2010) [50]	USA	52,75	164,55	NA	no onset of menarche by age 15 years	absence of three consecutive menstrual cycles in the past year	interval between menses more 35 days and less than 90 day in the past year
Mudd, Fornetti and Pivarnik (2007) [34]	USA	57,2 ± 5.6	165,1 ± 6.5	NA (collegiate athletic association division I university)	NA	0 to 3 menstrual cycles per year	4-9 cycles per year
Tenforde et al. (2017) [33]	USA	NA	NA	NA	non-appearance of menarche up to 16 years of age	less than 6 periods per year	6-9 periods per year
Hutson et al. (2021) [51]	UK	NA	NA	NA	NA	no menses for at least the previous 90 days	less than nine periods over the previous 12 months
Muia et al. (2015) [52]	Kenya	48,5 ± 6,1	159,2 ± 5,8	NA	absence of menstrual cycle by age 15	no menstrual cycle for more than 90 continuous days after menarche	cycles longer than 45 days
Beckvid Henriksson, Schnell, and	Sweden	54,7 ± 1,0	169,8 ± 1,1	10,0 ± 0,7	menstrual bleeding never occurred	cessation of menstrual bleedings for more than 3 successive months	interval pattern exceeding 6 weeks

Hirschberg (2000) [53]							
Pollock et al. (2010) [32]	UK	52,8 ± 4,8	166,0 ± 1,0	13,4 ± 5,2	NA	0–3 cycles/year	4–9 cycles/year
Burrows et al. (2003) [54]	UK	57,3 ± 6	165,0 ± 6	8 ± 5	NA	0–3 cycles a year	0–9 cycle a year
Cobb et al. (2003) [55]	USA	58,1 ± 1,2	166,1 ± 1	NA	NA	ewer than 4 cycles in the past year	4–9 menstrual cycles per year
Dusek (2001) [39]	Croatia	NA	NA	18 ± 4	non-appearance of menarche up to 16 years of age	absence of menstruation for more than 3 months in the post-menarche period	NA
Tenforde et al. (2015) [56]	USA	55,0 ± 8,5	163,9 ± 7,2	NA	non-appearance of menarche up to 16 years of age	less than 6 periods per year	6-9 periods per year
Rauh, Barrack and Nichols (2014) [57]	USA	57,9 ± 8	163,9 ± 6,6	NA	no onset of menses by age 15 years	cessation of menstrual cycles for ≥3 consecutive months in the past year	cessation of menstrual cycles for ≥3 consecutive months in the past year
Gibson et al. (2000) [44]	UK	56,4	164	NA	NA	0–3 cycles/year	4–9 cycles/year
Walsh, Crowell and Merenstein (2020) [58]	USA	59,0 ± 3,8	167,9 ± 5,3	NA	NA	NA	NA
		72,8 ± 8,6	176,3 ± 6,6	NA	NA	NA	NA
Dimitriou et al. (2014) [45]	UK	61,2 ± 1,8	171,8 ± 5,5	15,3 ± 4,1	NA	0-3 cycles per year	4-9 cycles per year
Coste et al. (2011) [59]	France	55,0 ± 5,8	164,4 ± 4,9	15,1 ± 3,9	NA	NA	NA

Schtscherbyna et al. (2009) [43]	Brazil	58,3 ± 6,2	164,0 ± 7	13,9 ± 3,6	absence of menstrual blood flow at age 16 y or, in the absence of pubertal development, at age 14 y	an absence of menstrual blood flow for 6 mo or for a period equal to the total of three previous menstrual cycles	menstrual cycles longer than 35 days
Tsukahara et al. (2021) [60]	Japan	54,2 ± 4,8	163,5 ± 4,9	NA	menarche having not occurred by the age of 15 years in the absence of secondary sexual characteristics or by the age of 16 years in the presence of normal growth and secondary sexual characteristics	menstrual cycle cessation of more than 3 months after regular menses	menstrual cycle duration of more than 35 days with 4–9 menstrual cycles over the past year
Sygo et al. (2018) [61]	Canada	60,4 ± 4,3	167,0 ± 6	15 ± 6,2	NA	NA	NA
Robbeson, Havemann-Nel and Wright (2013) [62]	South Africa	60,2	171	NA	NA	the absence of three or more consecutive (~ 90 days) menstrual cycles	menstrual periods at intervals greater than 35 days
Nattiv et al. (2013) [41]	USA	57,7 ± 1,6	166,9 ± 1,3	NA	NA	NA	NA
Feldmann et al. (2011) [63]	USA	NA	NA	NA	NA	more than 90 days since LMP or having missed 3 consecutive cycles in the 12 months preceding the study.	less than 9 periods/ year
Hoch, Stavrakos and Schimke (2007) [64]	USA	NA	NA	NA	NA	NA	NA
Duckham et al. (2015) [47]	UK	54,8 ± 5,1	167 ± 5	12,6 ± 4,5	NA	NA	less than 9 periods/ year
	UK	55,4	167	12,9	NA	NA	less than 9 periods/ year

Duckham et al. (2013) [65]							
Raymond-Barker, Petroczi and Quested (2007) [66]	UK	58,9 ± 6,79	164,38 ± 6,36	8,96 ± 6,36	lack of menarche	absence of more than 3 periods	irregular periods
Haakonssen et al. (2014) [46]	Australia	58,4 ± 5,9	170,0 ± 7	NA	NA	menstrual cycle had ceased for more than 3 months in the past year	NA
**Other individual sports**							
Trutschnigg et al. (2008) [74]	Canada	58.73 ± 7.92	164.8 ± 8.5	11.57 ± 4.56	NA	no menstrual cycle for 90 days or more	cycles longer than 35 days
Tenforde et al. (2017) [33]	USA	NA	NA	NA	non-appearance of menarche up to 16 years of age	less than 6 periods per year	6-9 periods per year
Egan et al. (2003) [37]	Sweden, USA, Canada national teams	53.7 ± 5.8	164.0 ± 0.05	13.7 ± 1.4	non-appearance of menarche up to 15 years of age	three or less menstruations per year	cycles of between 45 and 90 days
Mudd, Fornetti and Pivarnik (2007) [34]	USA	58.6 ± 8.1	157.9 ± 6.3	NA	NA	0 to 3 menstrual cycles per year	4-9 cycles per year
Corujeira et al. (2012) [73]	Portugal	NA	NA	18	NA	NA	NA
Helge and Kanstrup (2002) [72]	Denmark	165	53,7 ± 6,0	23,5 ± 2,4	NA	a menstrual cycle of more than 90 days	menstrual cycle of 36–90 days
	China		165,1 ± 6,1	28			

Meng et al. (2020) [35]		50,7 ± 5,9			the absence of menarche by the age of 15 years	the absence of menses for at least 3months	menstrual cycles of longer than 35 days in duration
Roupas et al. (2014) [68]	France	52,0 ± 4,8	NA	40,8 ± 13,9	the absence of menarche by the age of 15 years	bsence of menstruation for 3 months	presence of menstrual interval of more than 35 days,with four to nine periods in the past year
Maïmoun et al. (2013) [67]	France	52,4 ± 4,7	166,9 ± 5,5	41,4 ± 13,8	absence of menstruation in girls above 15 years	absence of menstruation for 3 months	menstrual interval of more than 35 days, with 4–9 periods in the past year
Salbach et al. (2007) [69]	Germany	NA	161 ± 9	20.9 ± 7.3	NA	NA	NA
Muñoz et al. (2004) [70]	Spain	48 ± 7	161,8 ± 6	20	NA	NA	NA
Czajkowska et al. (2019) [71]	Poland	NA	NA	12	NA	menstrual cycles <3 months long	menstrual cycles 3-6 months long
Klinkowski et al. (2008) [30]	Germany	49,7 ± 8,4	164 ± 7	18,9	menstruation either failed to occur until the age of 15	absent for the last three months	NA
Klentrou and Plyley (2003) [31]	Canada, Greek	49,7± 1,7	167,3 ± 1,5	22,5	NA	NA	NA
Di Cagno et al. (2012) [36]	Italy	49.5 ± 5,1	170 ± 1	30,7 ± 14,1	NA	NA	NA
Helge and Kanstrup (2002) [72]	Denmark	54,5 ± 5,8	165	21,0 ± 5,1	NA	a menstrual cycle of more than 90 days	menstrual cycle of 36–90 days
Coelho et al. (2013) [75]	Brazil	52,28 ± 7,74	159,5 ± 6,06	10,62 ± 3,46	NA	NA	NA
**Winter sports**							
	USA		165.6 ± 0.1	NA		less than 3 periods per year	

Meyer et al. (2004) [76]		63.0 ± 6.5			no menses till the age of 16 years		three to six periods per year
